# Metabolism of fatty acids in follicular cells, oocytes, and blastocysts

**DOI:** 10.1530/RAF-21-0123

**Published:** 2022-04-29

**Authors:** Meihong Shi, Marc-André Sirard

**Affiliations:** 1 Centre de Recherche en Reproduction, Développement et Santé Intergénérationnelle, Département des Sciences Animales, Faculté des Sciences de l’Agriculture et de l’Alimentation, Pavillon INAF, Université Laval, Québec, Québec, Canada

**Keywords:** fatty acids, oocyte, granulosa cells, cumulus cells, blastocyst

## Abstract

Fatty acids (FA) are one of the substrates that can be oxidized for energy production. The blood concentration of all types of FA varies according to different nutrition conditions, and follicular fluid levels are generally in line with serum levels. Elevated levels of FA, especially non-esterified fatty acids (NEFA), are commonly found in females with metabolic issues, which are often related to subfertility in many species including humans, pigs, cattle, and mice. Long-time exposure to an excessive quantity of fatty acids impairs the cell structure and functions causing injuries in tissues and organs, resulting in lipotoxicity and eventually hampering health and fertility. High levels of saturated NEFA can have detrimental effects on granulosa cells, oocyte quality, and embryo development. Although the harmful effects of FA are established in reproductive tissues, how granulosa cells and cumulus cells respond and cooperate with oocytes when exposed to NEFA requires further understanding. This review provides a summary of the adverse impacts of exposure to NEFA during *in vitro* maturation on oocytes, follicular cells, and embryos. A comprehensive understanding of the effects of NEFA on oocytes *in vitro* would improve our understanding of the impacts of natural exposure *in vivo*.

**Lay summary:**

Exposure to excess FAs affects the health of eggs, early embryos, and children born from these. The way different cell types react to excess FAs has not been studied very extensively, especially in pigs which provide a good model to investigate the impact of nutrition on the ovaries in humans. This review also looks at the way cells surrounding the egg react to FAs to help our understanding of the impact of excess fatty acids on female fertility.

## Introduction

Female fertility relies heavily on ovarian function and the resulting oocyte quality. The oocyte, which oversees subsequent development, is a relatively dependent entity. Although it can be matured and fertilized *in vitro,* its development *in vivo* is dependent on the status of its microenvironment before fertilization, which includes the follicular cells and the follicular fluid. In the follicle, the developing oocyte is surrounded by cumulus cells attached to the mural granulosa cells, and the roles of these two types of granulosa cells in maintaining the quality of the oocyte are determinant. Cumulus cells are regarded as nutritive cells because they provide the oocyte with paracrine factors and metabolic substrates including pyruvate. Mural granulosa cells communicate with cumulus cells via cell junctions to exchange metabolites, growth factors, and signaling messengers. Besides, the follicular fluid (FF) serves as another substrate supplier and the amounts of nutrients in FF are dependent on those in the bloodstream. Therefore, the composition of the FF and the oocyte reflects the mother’s nutrition. The facts that mitochondria are the units responsible for energy production and that most mitochondria in an individual originate from the oocyte, and are therefore derived from the mother, highlight the influence of maternal nutrition on the oocyte and potentially on the energy metabolism of the offspring.

Fatty acid (FA) concentrations are affected by the diet and the lipolysis of stored lipids. The FA concentrations, especially non-esterified fatty acids (NEFA), which are also known as free fatty acids (FFA), are increased during both over- and under-nutrition conditions, and the elevated levels are associated with low embryo development in humans, cattle, and pigs (Leroy *et al.* 2005, Hoving *et al.* 2012, Valckx *et al.* 2014*b*). Some of the excess FAs are stored in lipid droplets, some exist as FFA, some are transferred into mitochondria and β-oxidized for energy production, and some remain in the cytosol and either undergo peroxidation and generate ROS or affect the endoplasmic reticulum (ER) calcium store and cause ER stress. The level of toxicity for cells strongly depends on the type of FA, saturated or unsaturated, to which cells are exposed.

In recent years, more studies focused on the effects of elevated levels of NEFA on granulosa cells, cumulus cells, oocytes, and early embryo development in various species (Mu *et al.* 2001, Leroy *et al.* 2005, Aardema *et al.* 2011, Van Hoeck *et al.* 2011, Aardema *et al.* 2013, Desmet *et al.* 2016, Sutton-McDowall *et al.* 2016, Aardema *et al.* 2017, Itami *et al.* 2018, Ogawa *et al.* 2018, Shi & Sirard 2021*a*). Additionally, ovarian dysfunctions induced by high fat diets were also investigated in humans (Chavarro *et al.* 2007), mice (Yi *et al.* 2012) and cattle (Zachut *et al.* 2010). Previous reviews paid more attention to species such as human, cattle, and mice. Because of some similarities including a very comparable digestive physiology and metabolism (monogastric) and an approximate length of IVM in humans and pigs, the pig is an excellent model to study the impacts of nutrition on ovarian function in humans without all the ethical limitations. Considering that the relationships between the different follicular cell types under the influence of FA have not been studied very extensively in the past, this review presents our revised understanding of the effects of elevated NEFA on female reproductive success and also of the protective functions of granulosa and cumulus cells on the oocytes of pigs and other mammals.

## Energy metabolism in cumulus–oocyte complexes

The oocyte is the female germ cell involved in reproduction, and it is the source of life in mammals. Its development and the initial days of the subsequent embryo development are energy-consuming processes. As opposed to somatic cells, oocytes and early embryos need to metabolize not only the principal energy sources from exogenous nutrients but also energy sources from intracellular stores (Sturmey & Leese 2003). Substrates stored in oocytes can support the energy requirements of the early days of embryo development since sufficient and appropriate energy metabolism plays a critical role in maintaining developmental competence. *In vitro* matured oocytes and *in vitro* produced embryos have reduced developmental competence and they have reduced capacity to utilize glucose, suggesting that energy deficiency is an indicator of lower oocyte and embryo qualities (Krisher *et al.* 2007). When considering the metabolism of the oocyte, it is important to consider the interactions between the oocyte, cumulus cells, and granulosa cells because these cells produce and exchange metabolites during development.

### Carbohydrate metabolism

Glucose is metabolized via glycolysis, the pentose phosphate pathway (PPP), and tricarboxylic acid (TCA) in oocytes. Metabolism of pyruvate, glutamine, and glycine by the Krebs cycle increases during *in vitro* maturation, indicating that oxidative metabolism plays an essential role in providing energy for oocyte maturation. Compared to immature cumulus–oocyte complexes (COCs), mature COCs consume higher amounts of substrates and store more energy for fertilization and subsequent early embryo development. Moreover, during all the stages of development from immature oocyte to expanded blastocyst, most of the ATP (about 95%) is generated by oxidative metabolism (Sturmey & Leese 2003). Different from mouse and cow oocytes, which prefer to use pyruvate produced by and transferred from cumulus cells, porcine oocytes prefer to utilize glucose as their primary substrate for energy production, and the pathways of glucose metabolism are also different between oocytes matured *in vivo* and *in vitro* (Krisher *et al.* 2007).

### Lipid metabolism

In addition to playing important roles in many biological functions including biological membrane construction, cell–cell interactions, steroid synthesis, and signaling, lipids also act as a source of nutrients. Decreased triglyceride, phospholipids, cholesterol, and total lipids during oocyte maturation suggest the important role of lipid metabolism in oocyte maturation (Ferguson & Leese 1999, Sturmey & Leese 2003). FAs are utilized via β-oxidation in mitochondria, and the lipid–mitochondrial microstructures highlight the importance of lipids in generating ATP production. Considering one single molecule of FAs (such as palmitate) and glucose, the use of lipids yields substantially higher amounts of ATP compared to carbohydrates. Studies of genes involved in lipid metabolism were conducted in porcine and bovine ovaries (Uzbekova *et al.* 2015, Bertevello *et al.* 2018), and the different transcription patterns of genes related to FA metabolism in oocytes, theca cells, granulosa cells, and cumulus cells indicated that the different follicular compartments play different roles in lipid homeostasis.

The β-oxidation of FA was reduced in both cumulus cells and oocytes matured *in vitro* compared to cumulus cells and oocytes matured *in vivo* in pig (Yuan *et al.* 2011) and mouse (Dunning *et al.* 2014). *In vitro* maturated oocytes display a lower developmental competence, and lipid oxidation seems to be involved in creating this difference.

### Lipids are required for energy production during oocyte maturation

FAs, stored in lipid droplets as triglycerides, are metabolized by β-oxidation and the TCA cycle in mitochondria. Closed location of mitochondria and lipid droplets in oocytes makes them a ‘metabolic unit’, which ensures a steady and readily available supply for lipid oxidation processes (Kruip *et al.* 1983, Sturmey *et al.* 2006). The co-localization of mitochondria and surrounding lipid droplets (with a distance of 6–10 nm) reinforces the important role of lipid metabolism during oocyte maturation (Sturmey *et al.* 2006). The essential role of lipid β-oxidation can be also demonstrated by its inhibition and activation since the alteration of β-oxidation can lead to impairment in oocyte maturation and development (Ferguson & Leese 2006, Dunning *et al.* 2011). A study investigating the effects of FA on granulosa cells showed that the inhibition of FA oxidation inhibits proliferation and progesterone secretion in bovine granulosa cells (Elis *et al.* 2015). Therefore, moderate β-oxidation that provides enough ATP but without excess production of ROS during maturation is very important for oocyte developmental competence. However, the degree of dependence on lipid metabolism in oocytes varies between species. Compared to bovine and mouse oocytes, pig oocytes contain more lipids and a higher number of lipid droplets, and partial inhibition of FA β-oxidation can inhibit porcine oocyte maturation while the inhibition of FA β-oxidation do not impair mouse and bovine oocyte maturation since they are capable to use carbohydrate metabolism to compensate for a deficiency in energy produced by FA β-oxidation (Paczkowski *et al.* 2013). This study suggests that the lipid metabolism during oocyte maturation is more important in the species of which oocytes contain more lipids (McEvoy *et al.* 2000).

### Balance between lipid oxidation and glucose consumption

We know that oocytes metabolize carbohydrates (pyruvate and lactate) and lipids (free fatty acids) to generate energy to maintain and support their biological development; however, these metabolic processes produce side products such as reactive oxygen species (ROS). Basal ROS production is an unavoidable natural mechanism in cells, but prolonged and high ROS production can cause oxidative stress which induces apoptosis. The ‘quiet embryo hypothesis’ described by Leese (2002) stipulates that having enough energy and maintaining a minimum level of ROS is optimal for oocyte and early embryo development. In such conditions, the activity of some oocyte and embryo mitochondria is ‘shut down’ to maintain the quiet status. Oocytes and cleavage-stage embryos have lower oxygen consumption than blastocyst, suggestion ‘shut down’ metabolic activity (Sturmey & Leese 2003). Prolonged exposure to high levels of extracellular lipid in cells resulted in excess peroxidation of free FA, leading to the production of excess ROS and toxic lipid peroxides, which together are known as lipotoxicity (Igosheva *et al.* 2010). Therefore, the balanced metabolism of pyruvate and FA, which keeps a low level of ROS, is important for oocyte quality and developmental competence.

## Lipid profiles in follicular cells

The lipid content of oocytes varies from species to species. Before maturation, pig oocytes contain 74 ng of triglycerides out of a total lipid content of 161 ng, and bovine oocytes contain 23 ng of triglycerides out of the total lipid content of 63 ng (McEvoy *et al.* 2000). Oocytes that contain a large amount of intracellular lipids are more difficult to cryopreserve because they are prone to cryoinjury and are more sensitive to temperature changes. The reasons why the lipid content varies between species are unknown, but there is one hypothesis that the species with a high lipid content in oocytes have a longer duration from ovulation to implantation because the stored lipids can act as endogenous energy reserves to maintain their basic needs until implantation (Sturmey *et al.* 2009, Bradley & Swann 2019). More studies are needed to explore the mechanisms responsible for the differences in lipid contents between species.

### Dynamics of lipid profiles in oocytes

The analysis of FA profiles in pigs showed that the proportions of free saturated FA are 46, 40, and 37% in oocytes, FF, and serum, respectively; and the proportions of free mono-unsaturated FA are 27, 36, and 46% in oocytes, FF, and serum, respectively; and the proportions of free polyunsaturated FA are 27, 24, and 15% in oocytes, FF, and serum, respectively (Yao *et al.* 1980, Homa *et al.* 1986). The ratios of total free FA in oocytes are closer to those in FF, suggesting that the free FA content is derived directly from the FF rather than from the serum. It is important to notice that lipid metabolism is a dynamic process, and the composition of fatty acids is easily influenced by the maternal energetic state. Besides, palmitic acid (PA), oleic acid (OA), and stearic acid (SA) are the major FA of oocytes, FF, oviductal fluid, uterine fluid, and serum in pigs and cows, and they comprise approximately 60–80% of the total FA (Yao *et al.* 1980, Homa *et al.* 1986).

It is well known that organisms need nutrients to produce energy to maintain their biological functions besides surviving. There are differences in nutrient needs and energy storage in domestic animals between winter and summer. The FA composition of phospholipids in FF, granulosa cells, and oocytes in cows changes from summer to winter. In summer, there is a relatively higher level of saturated FA than that in winter (Zeron *et al.* 2001). Seasonal changes were associated with cow fertility and oocyte quality both *in vivo* and *in vitro* studies (Ryan *et al.* 1993), and the varying FA compositions between seasons may explain the decreased fertility in summer since the saturated FAs have more detrimental effects than unsaturated FAs on oocyte quality and development. Similarly, lower fertility, with decreased follicle and oocyte development, was also observed in pigs according to season (Bertoldo *et al.* 2010). Therefore, it is interesting to investigate whether there is a seasonal difference in the FA composition of porcine oocytes and verify the relationship between the FA composition and oocyte quality.

In addition to seasonal changes, lipid profiles also vary during follicle and oocyte development. Yao *et al.* found that the composition of free and esterified FA in porcine FF was different at the different follicle development stages (1980). The NEFA content decreased during follicular development, and the large follicles had the lowest level of NEFA which was similar to the serum level. In porcine oocytes, the amount of triglycerides, which is the major constituent of total lipid, decreased during maturation but remained stable until blastocyst development (Sturmey & Leese 2003). A similar pattern was observed during cow oocyte maturation and early embryo development (Ferguson & Leese 1999). Besides, another study using Nile Red fluorescent probe reported that the levels of phospholipids and cholesterol in lipid droplets also significantly decreased from immature to mature porcine oocytes (Romek *et al.* 2011). Although there was no decrease in triglycerides and phospholipids from the zygote to the morula stage, decreases were observed from morula to blastocyst and from blastocyst to hatched blastocyst (Romek *et al.* 2011). The divergences between these results and the results mentioned above can be explained by the different methods used to measure the triglyceride content. The decreased lipid content during porcine oocyte and early embryo development highlights the metabolic role of lipids, but it is necessary to consider the roles of other supplements, the serum supplemented in the IVM medium increases the lipid content in oocytes for example, that can mask these changes in endogenous reserves.

### Varied levels of FA in abnormal metabolic conditions

It was mentioned before that the FA content of the follicular fluid may influence the relative FA content of the oocyte, and the concentrations of NEFA in the follicular fluid are dependent on their concentrations in the serum, which are easily influenced by the nutrition status (Leroy *et al.* 2005, Jungheim *et al.* 2010, Aardema *et al.* 2013). Therefore, dietary FA can disrupt the original lipid profile of the FF and thus of the oocyte, and these maternal changes are associated with oocyte quality, subsequent development, and metabolic diseases in offspring which may cross generations (Valckx *et al.* 2014*a*, Chambers *et al.* 2016). Besides, abnormal nutrition conditions such as diabetes in women and negative energy balance in cows and pigs were associated with elevated levels of NEFA (Leroy *et al.* 2005, Hoving *et al.* 2012, Valckx *et al.* 2014*b*).

Negative energy balance (NEB) is a metabolic status achieved in the period after calving when the food intake does not satisfy the high energy demand of milk production in cattle. During this period, cows suffer from metabolic and endocrine issues, resulting in ovarian problems, poor oocyte quality, and reduced reproductive performance. Another obvious change is the two- to three-fold increase in the serum and follicular fluid NEFA levels in postpartum cows compared to the levels of prepartum cows (Leroy *et al.* 2005). There is a similar problem in sows, where weight loss during lactation has negative effects on ovulation rates, oocyte quality, embryo survival, and litter development (Vinsky *et al.* 2006). Elevated levels of follicular NEFA were also observed in sows experiencing lactation weight loss (Hoving *et al.* 2012).

Meanwhile, elevated levels of NEFA were also observed in women with high BMI values and obesity (Valckx *et al.* 2014*b*); and FA concentrations in FF and infertility rates were positively correlated to increased BMI. A high-fat diet can also modify NEFA levels, and mice fed a high-fat diet had a higher concentration of NEFA in the serum (Tian *et al.* 2016). Besides, the high-fat diet increased the number of abnormal mitochondria and induced spindle and chromosome alignment defects in mouse oocytes (Luzzo *et al.* 2012). Besides, studies in mice demonstrated that diets rich in specific lipids could alter the lipid profile of ovarian cells, and along with lipotoxicity responses, impaired fertilization (Wakefield *et al.* 2008, Wu *et al.* 2010). In mouse and rat studies, metabolism dysfunctions induced by high-fat diets could be transmitted to next generations (Dunn & Bale 2009, Chambers *et al.* 2016).

The NEFA concentrations and composition profiles in the serum and FF of cows, humans, and pigs either with normal or abnormal nutritional status are listed in Table 1. There are differences and similarities between the NEFA concentrations of serum and FF of different species. The physiological concentrations of NEFA in bovine serum and FF are similar, but the concentration of NEFA in serum was increased with a higher level than that in FF under NEB conditions (Leroy *et al.* 2005). The NEFA concentration in human FF is lower than in the serum, and the altered levels under obesity are almost the same in both FF and serum (Igosheva *et al.* 2010, Valckx *et al.* 2012, 2014*b*). However, the concentration of NEFA in pig FF is slightly higher in the serum (Yao *et al.* 1980). No study measured the concentration of NEFA in the FF of sows experiencing NEB. Most differences can be explained as species differences, but the large variation in pig serum may be due to breed differences. Besides, the reflection of an increase in the total NEFA levels from serum in the FF demonstrates their close relationship.

**Table 1:** NEFA concentrations and composition profiles of physiological and pathological status of bovine, human, and porcine.

	Bovine		Human		Porcine	
	Serum	FF	Serum	FF	Serum	FF
Physiological NEFA						
Concentrations (μM)	100–300	100–300	600–700	200	200–400; 600–800	600–900
Composition profile						
PA	17%	22%	–	23%	20%	27%
SA	28%	15%	–	9%	17%	12%
OA	22%	30%	–	33%	44%	33%
Pathological NEFA						
Concentrations (μM)	400–1200	200–600	700–900	300	600–1200	–
Composition profile						
PA	21%	22%	-	22%	–	–
SA	24%	13%	-	13%	–	–
OA	35%	37%	-	37%	–	–
References	Leroy* et al*. (2005)	Leroy *et al*. (2005)	Igosheva *et al*. (2010), Valckx *et al*. (2012, 2014*b*)	Valckx *et al*. (2014*b*)	Yao *et al*. (1980), Hoving *et al.* (2012)	Yao *et al*. (1980)

The conditions mentioned above reveal that NEFA levels may play important roles in regulating oocyte quality and development, which could have impacts on progeny since the maternal nutrition status has been highly correlated with metabolic disorders in offspring (Valckx *et al.* 2014*a*, Chambers *et al.* 2016).

## Oocyte maturation and its subsequent development are sensitive to the lipid environment

### Lipid uptake and storage

As mentioned above, lipid storage undergoes dramatic changes during oocyte maturation and early embryo development, varies seasonally, or differs according to the nutritional status. But whether other supplementations to *in vitro* culture media could increase lipid storage needs to be addressed separately. Oocytes matured in the presence of 10% fetal calf serum had more lipid stored, including triglycerides and cholesterol, compared to oocytes cultured in serum-free medium (Yang *et al.* 2010). It should be noted that the serum FA composition changes under specific metabolic stress, and these changes are reflected in the FF, oocyte, and ovarian cells but to different extents. The differentially expressed lipid metabolism-related genes are corresponding to the different functions and the differential lipid profile of follicular cells and oocytes (Uzbekova *et al.* 2015).

Besides, the supplementation of SA or PA decreased lipid accumulation in bovine oocytes, but a high level of OA or linoleic acid could increase neutral lipid accumulation (Aardema *et al.* 2011, Carro *et al.* 2013). When mouse oocytes were exposed to human FF enriched in triglycerides and FFA during maturation, the neutral lipid content increased (Yang *et al.* 2012). Also, when the culture medium of pig COCs was supplemented with a mix of NEFAs (PA, SA, and OA) or SA alone, the lipid content of the cumulus cells reflected the altered composition of the medium, but the lipid content of oocytes was not affected (Ogawa *et al.* 2018, Pawlak *et al.* 2020). It is similar to bovine where exposure to a high level of the mix of NEFAs (PA, SA, and OA) during IVM resulted in a massive lipid accumulation in cumulus cells but not in the oocyte (Aardema *et al.* 2013). However, supplementation with PA was able to increase the lipid content in porcine oocytes (Shibahara *et al.* 2020). Although differences exist between species and variations also depend on concentrations used, it can be speculated that cumulus cells play the role of accumulating extra FA when exposed to the high level of the mix of NEFAs and the presence of unsaturated FFA promotes this accumulation process.

### Saturated FFA have detrimental effects

The addition of extra NEFAs to the culture medium not only changed the lipid content but long-term excessive lipid exposure could also result in detrimental effects, which are collectively referred to as lipotoxicity (Igosheva *et al.* 2010). Studies in bovine, the most studied model, showed that an elevated level of NEFA reduced cell viability and steroidogenesis and increased apoptosis of granulosa, cumulus, and theca cells (Leroy *et al.* 2005, Vanholder *et al.* 2006, Wu *et al.* 2012, Sutton-McDowall *et al.* 2016, Aardema *et al.* 2017, Sharma *et al.* 2019, Wang *et al.* 2020). Besides, high levels of NEFA in the IVM medium resulted in delayed maturation, low fertilization, and decreased blastocyst formation. The blastocysts displayed lower cell numbers, higher apoptotic ratios, and aberrant metabolism (Van Hoeck *et al.* 2011, Desmet *et al.* 2016).

Supplementation of NEFA also had negative effects on porcine granulosa cell proliferation and viability and increased the lipid content in granulosa and cumulus cells (Ogawa *et al.* 2018, Pawlak *et al.* 2020, Shibahara *et al.* 2020). Besides, the high level of NEFA altered transcriptomic pattern which is enriched in pathways related to metabolism, inflammation, and impaired mitochondrial function in granulosa cells (Shi & Sirard 2021*a*). After ovulation, the epithelial–mesenchymal transition (EMT) of granulosa cells to luteal cells occurs, which is associated with changes in estradiol and progesterone production. Inhibition of EMT and altered steroidogenesis in response to high levels of NEFA partly explain the anovulation and impaired follicle development observed in pathological nutritional status (Foong *et al.* 2006, Chaput & Sirard 2020, Shi & Sirard 2021*a*). Moreover, supplementation with NEFA during IVM also decreased the blastocyst formation (Shibahara *et al.* 2020, Shi & Sirard 2021*c*). However, another study in porcine found that supplementation with NEFA-rich FF or NEFA was not harmful to the oocyte developmental competence (Ogawa *et al.* 2018). These controversial results can be explained by the different concentrations used. The concentration of that increased blastocyst formation was less than half of the actual level in FF and much lower than the pathological level, and the other factors in the supplemented FF that do not exist in control (only with BSA) may be responsible for this beneficial effect.

Similarly, the survival of human granulosa cells was reduced under elevated SA and PA concentrations (Mu *et al.* 2001). Human embryos originating from oocytes from follicles with a higher percentage of saturated FA, especially PA and SA, failed to cleave (O’Gorman *et al.* 2013). Moreover, the IVF outcome of obese patients could be improved if the oocytes were from a donor with normal BMI; however, oocytes from a mother on a high-fat diet suffered the same defects and failed to develop into blastocysts even if they were transferred to a surrogate with normal BMI (Jungheim *et al.* 2013). It could be concluded that the high-fat environment has stronger effects at the oocyte level. In mice, the supplementation of NEFA reduced follicular antrum formation, increased progesterone synthesis, induced apoptosis in granulosa cells, and impaired oocyte maturation and development (Valckx *et al.* 2014*a*, Chen *et al.* 2019).

Further studies aimed at understanding the mechanisms involved in the effects of elevated NEFA via analyses of transcriptome and DNA methylation. The methylation pattern of the maternal imprinted genes and several metabolism-related genes were altered in oocytes from both obese mice and their offspring (Ge *et al.* 2014). In bovine, blastocysts originating from oocytes exposed to elevated NEFA had different transcriptomic patterns in pathways related to lipid and carbohydrate metabolism and cell death (Van Hoeck *et al.* 2015). Similarly to gene expression, the DNA methylation pattern of blastocysts originating from oocytes exposed to NEFA was altered in pathways involved in lipid and carbohydrate metabolism, cell death, immune response, and metabolic disorders (Desmet *et al.* 2016). In pigs, transcriptome and DNA methylation patterns of blastocysts from oocytes exposed to NEFA were also altered, and affected pathways were mainly related to metabolism and inflammation (Shi & Sirard 2021*c*). Besides, transcriptomic analysis of GCs cocultured with COCs showed that metabolism, inflammatory responses, mitochondrial dysfunctions, and the EMT were all affected by the elevated level of NEFA (Shi & Sirard 2021*a*). The differences and similarities in the effects of high levels of NEFA in different species are listed in Table 2.

**Table 2:** NEFA effects on biological functions in different cell types of different species.

Species/exposure	Cell type	Affected biological functions	Reference
Humans			
*In vitro*	GCs	Cell viability, cell proliferation, apoptosis, steroidogenesis, fatty acid metabolism	Mu *et al.* (2001)
Bovine			
*In vitro*	GCs, CCs, TCs	Cell proliferation, apoptosis, steroidogenesis, ROS production, ER stress, EMT	Leroy *et al.* (2005), Vanholder *et al.* (2006), Wu *et al.* (2012), Aardema *et al.* (2013), Sutton-McDowall *et al.* (2016), Sharma *et al.* (2019), Wang *et al.* (2020), Yenuganti *et al.* (2021)
	Oocyte	Maturation, fertilization, development competence, apoptosis	Leroy *et al.* (2005), Aardema *et al.* (2011), Carro *et al.* (2013)
	Blastocyst	Blastocyst formation, blastocyst formation, energy metabolism, ER stress, inflammation, transcriptome, DNA methylation pattern	Leroy *et al.* (2005), Van Hoeck *et al.* (2011, 2013, 2015), Desmet *et al.* (2016), Sutton-McDowall *et al.* (2016)
*In vivo*	GCs, CCs	Lipid storage, lipid profile	Zachut *et al.* (2010), Aardema *et al.* (2013)
Porcine			
*In vitro*	GCs, CCs	Lipid storage, cell proliferation, cell viability, metabolism, EMT	Ogawa *et al.* (2018), Pawlak *et al.* (2020), Shibahara *et al.* (2020)
	Oocyte	Maturation, mitochondrial functions, lipid storage, development competence	Itami *et al.* (2018), Ogawa *et al.* (2018), Shibahara *et al.* (2020)
	Blastocyst	Lipid storage, development competence, metabolism, inflammation, transcriptome, DNA methylation pattern	Pawlak *et al.* (2020), Shibahara *et al.* (2020), Shi and Sirard (2021*b*)
*In vivo*	Ovary	Follicle development, apoptosis, antioxidants production	Xu *et al.* (2016)
Mouse			
*In vitro*	GCs	Steroidogenesis, cell viability, apoptosis	Valckx *et al.* (2014*a*), Chen *et al.* (2019)
	Oocyte	Lipid storage, ER stress, maturation, development ability	Valckx *et al.* (2014*a*), Yang *et al.* (2012)
	Blastocyst	Development ability, ER stress, lipid storage, mitochondrial superoxide level	Valckx *et al.* (2014*a*), Yousif (2019)
*In vivo*	GCs, CCs, oocyte, blastocyst	Mitochondrial activity, ROS production, apoptosis, ER stress, ovulation, fertilization, blastocyst formation	Igosheva *et al.* (2010), Wu *et al.* (2010)
Sheep			
*In vivo*	GCs, oocyte, blastocyst	Fatty acid composition, steroidogenesis, cell proliferation, embryo development	Wonnacott *et al.* (2010)

CCs, cumulus cells; EMT, epithelial–mesenchymal transition; ER, endoplasmic reticulum; GCs, granulosa cells; ROS, reactive oxygen species; TCs, theca cells.

It seems that different types of FA impair cell functions via different mechanisms. A high-fat diet or direct exposure to excessive FA induced mitochondrial abnormalities in oocytes that could be countered by antioxidants (Boots *et al.* 2016); however, antioxidants seemed useless to counter the injuries caused by PA in early embryo development (Nonogaki *et al.* 1994). In the case of PA, endoplasmic reticulum calcium ATPase pumps were negatively affected, and the ER stress inhibitor salubrinal® could reverse the impaired oocyte development induced by PA exposure (Wu *et al.* 2012). Elevated SA and NEFA levels upregulated redox genes in oocytes but decreased their relative expression in cumulus cells, indicating their different metabolism in oocytes and cumulus cells (Van Hoeck *et al.* 2013).

### Mono-unsaturated FA counteracts the adverse effects of saturated FA

Among the saturated FA, different types have different mechanisms to affect oocyte quality and development. Excess saturated FA undergo peroxidation, leading to increased apoptosis and reduced success of fertilization and development (Nonogaki *et al.* 1994, Itami *et al.* 2018). However, unsaturated FA have beneficial effects on fertility, and they can counteract the adverse effects of saturated FA. Linoleic acid and OA improved bovine and mouse embryo development in a dose-dependent manner, but a high dose impaired maturation (Marei *et al.* 2010, Aardema *et al.* 2011). Similarly, studies in cows found that supplementation with either linolenic or alpha-linolenic acid increased the corresponding FA in FF, granulosa cells, COCs, as well as improved the cleavage rate (Zachut *et al.* 2010). Besides, a diet supplemented with omega 3 and 6 affected the FA composition and development of granulosa cells, oocytes, and blastocysts in sheep (Wonnacott *et al.* 2010). Higher concentrations of total unsaturated FA are related to better follicle growth, oocyte quality, fertilization, and early embryo development. Unlike saturated FA, unsaturated FA is preferentially and more easily stored as triglycerides, and activation of the peroxisome proliferator-activated receptors (PPARs) by unsaturated FA can reduce lipotoxicity of saturated FA (Nolan & Larter 2009). Reduced lipotoxicity was demonstrated when supplementation of OA improved embryo morphology and blastocyst formation under exposure to PA and SA (Aardema *et al.* 2011, Valckx *et al.* 2014*a*). However, the ultimate effect of unsaturated FA, especially OA, was dependent on their concentration and the ratio of OA to other FA: a low concentration of OA and its low ratio to saturated FA may not have any beneficial effect.

Short-term changes in NEFA levels by fasting do change the composition of NEFA in serum, FF and cumulus cells, but does not change that of oocytes nor the subsequent developmental competence (Aardema *et al.* 2013). However, elevated NEFA levels *in vitro* changed the FA composition profiles of all cell types and hampered oocyte quality and its developmental competence (Vanholder *et al.* 2006, Valckx *et al.* 2014*a*, Van Hoeck *et al.* 2015, Desmet *et al.* 2016, Sutton-McDowall *et al.* 2016, Pawlak *et al.* 2020, Shibahara *et al.* 2020, Wang *et al.* 2020, Shi & Sirard 2021*b*). Distinctions between *in vivo* and *in vitro* studies could be explained by the different environments since the *in vivo* environment is more homeostatically precise and dynamic under the regulation of all the follicular cells and the FF. Additionally, storage and utilization by other tissues and cells, their interactions *in vivo*, and other factors might be responsible for these differences. Therefore, when exploring the effects of NEFA on oocytes, we need to consider the role of the other ovarian cell types.

## Protective functions of cumulus and granulosa cells exposed to NEFA

The follicular environment and the orchestrated crosstalk between somatic cells and the oocyte are essential conditions for the oocyte to achieve competence for fertilization. In antral follicles, specialized granulosa cells which surround the oocyte are named cumulus cells. Cumulus cells and the connected mural granulosa cells supply nutrients to the oocyte. Gap junctions between cumulus cells and the oocyte or mural granulosa cells allow bidirectional paracrine signaling, including signals for the regulation of chromatin remodeling and RNA synthesis in the oocyte. Besides, the utilization of glucose by cumulus cells is the way to provide pyruvate to the oocyte, which has a low capacity to utilize glucose and support maturation. Cumulus cells are involved in providing FA to oocytes and affect their lipid metabolism. The removal of cumulus cells before IVM decreased bovine oocyte developmental competence, and this suboptimal cytoplasmic maturation was associated with affected lipid metabolism probably via increased lipolysis and FA oxidation in the oocyte (Aardema *et al.* 2013, Auclair *et al.* 2013, Lolicato *et al.* 2015, Aardema *et al.* 2017). Therefore, it is believed that cumulus and granulosa cells have protective functions to ensure proper oocyte development, especially during exposure to excess NEFA.

### Lipid storage

FAs in circulation are transported into cells via either FA transporter proteins or by direct diffusion through the lipid bilayer. The transportation of follicular fluid FA into cumulus cells and oocytes is with the help of albumin or lipoproteins. However, cumulus cells and oocytes have different abilities to uptake and utilize FAs and lipids, as demonstrated by the different expression patterns of genes related to lipid metabolism in cumulus cells and oocytes (Uzbekova *et al.* 2015). Whether these cells prefer to use their lipid stores or the lipids from the extracellular milieu and how they interact with each other under lipid stress are not fully clear.

Bovine oocytes matured *in vitro* in the absence of cumulus cells have fewer lipid content, which implies that denuded oocytes may use more stored lipid (Auclair *et al.* 2013). With the exposure to NEFAs, there was a higher level of FA incorporation when the oocytes were maturated without cumulus cells (Lolicato *et al.* 2015). Besides, the oocytes matured without cumulus cells were more vulnerable when they were exposed to saturated FA (Aardema *et al.* 2017). These studies stress the important roles of cumulus cells in regulating the lipid content in oocyte and in protecting oocytes from the damages of excess saturated FA. In the presence of excess NEFA, granulosa cells, cumulus cells, and oocytes accumulated lipids (Yang *et al.* 2012, Carro *et al.* 2013, Ogawa *et al.* 2018, Pawlak *et al.* 2020, Shibahara *et al.* 2020). Besides, lipid storage in response to exposure to mono-unsaturated FA is one possible mechanism to explain the counteracting effects of OA on the negative effects of other saturated FA. The more lipid stored, the fewer free FA can undergo peroxidation and less ROS are generated. Therefore, lipid storage in granulosa cells and cumulus cells can decrease the amount of NEFA that oocytes are exposed to.

A cellular enzyme, stearoyl-CoA desaturase (SCD), can desaturate saturated FA into mono-unsaturated FA. The inhibition of SCD decreased lipid storage in bovine cumulus cells and impaired blastocyst formation, suggesting a protective role of cumulus cells that decrease the negative effects of NEFA on oocytes (Aardema *et al.* 2017). Unlike in bovine, SCD is expressed not only in porcine cumulus cells but also in oocytes and embryos at different stages (Lee *et al.* 2019, Pawlak *et al.* 2020). This might help porcine oocytes store FA as lipid droplets since mono-saturated FAs have more potential to promote lipid storage than other NEFA. Besides, the expression of SCD in porcine oocytes suggests that they may be more tolerant of high levels of NEFA because they can desaturate the saturated FA, and the high level of saturated FAs is more potentially toxic than the same level of unsaturated FA.

### Increased β-oxidation

In addition to being stored, FAs are oxidized for energy production in mitochondria or undergo peroxidation in the cytoplasm. Increased FA uptake via supplementation with L-carnitine or 5-aminoimidazole-4-carboxamide ribonucleotide (AICAR) can rescue cardiac mitochondria from lipotoxicity (Oyanagi *et al.* 2011), suggesting that an increased metabolism of FA is a kind of protection to decrease the excess FA available for peroxidation in the cytoplasm. Therefore, the oxidation of FA in cumulus cells and granulosa cells is another way to decrease the total amount of free NEFA in cells and decrease the lipotoxicity of NEFA to the oocyte.

### Interactions between oocytes and granulosa cells

The transfer of factors through gap junctions between granulosa and cumulus cells and between cumulus cells and the oocyte plays important roles in maintaining proper growth and development. Cumulus cells can protect the oocyte from ROS by regulating redox homeostasis and providing glutathione to the oocyte. Under exposure to elevated levels of saturated NEFAs, more ROS are produced and the redox regulation of cumulus cells is unable to supply enough protection to the oocyte from ROS, which was shown as the ROS accumulation in COCs and the deterioration of cumulus cells (Lolicato *et al.* 2015). Compared to blastocysts from COCs cultured without a monolayer of granulosa cells, blastocysts originated from cocultured COCs had anti-inflammatory responses to the exposure to high levels of NEFA, as demonstrated by downregulation of inflammatory factors including SP1 transcription factor (SP1), tumor necrosis factor (TNF), and nuclear factor kappa B (NFKB), and increase of anti-apoptosis factors such as BCL2 like 1 (BCL2L1) (Shi & Sirard 2021*b*). It can be speculated that some factors originating from cocultured granulosa cells can regulate inflammation. However, these factors have not been identified, and more studies are needed to find and verify them.

Mural granulosa cells and cumulus cells are barriers that protect oocytes from direct exposure to the hazardous environment. In the case of excess FAs, granulosa cells decrease the amount the oocyte is exposed to, either by storage and utilization like cumulus cells, or by activating response mechanisms including anti-inflammatory actions to make the oocyte healthier.

## Conclusions

**Figure 1.**
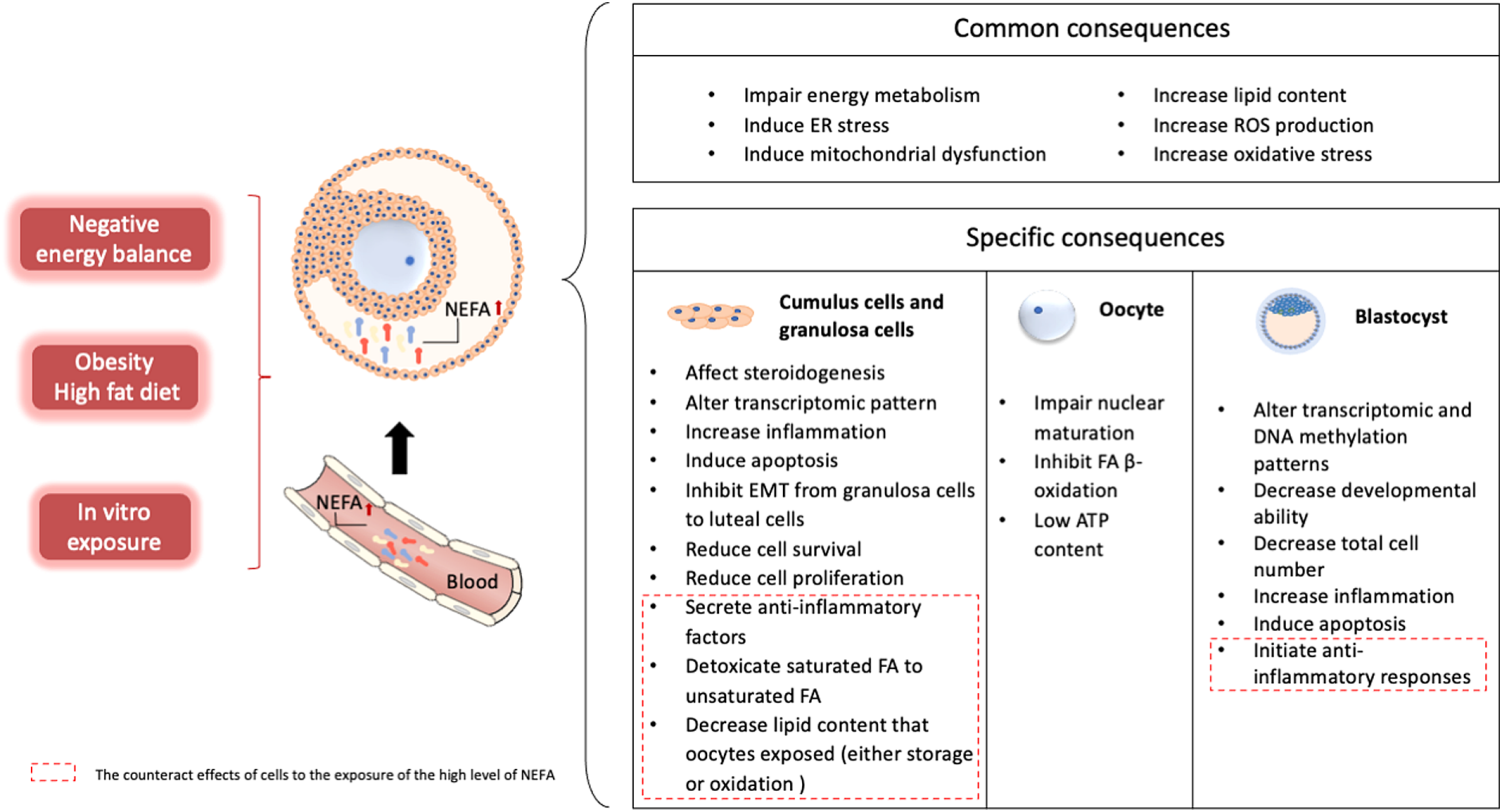
The effects of the high levels of NEFA in ovarian cells. Some pathological nutrition statuses including negative energy balance or obesity or high-fat diet and *in vitro* exposure cause elevated NEFA levels in serum and follicular fluid. Impaired energy metabolism, ER stress, mitochondrial dysfunction, increased lipid content, increased ROS production, and increased oxidative stress are the common consequences of high levels of NEFA on ovarian cells (including granulosa cells, cumulus cells, and theca cells), oocytes, and blastocysts. Besides, there are some specific consequences in different cell types. The cell survival, proliferation, and steroidogenesis of granulosa cells and cumulus cells are affected by exposure to the high levels of NEFA. Besides, the epithelial-mesenchymal transition of granulosa cells to luteal cells is also negatively affected, and the transcriptomic pattern was altered. In oocytes, the high level of NEFA impairs the nuclear maturation, inhibits FA β-oxidation, and decreases ATP content. Blastocysts originating from oocytes exposed to the high level of NEFA showed less total cell number and altered transcriptomic and DNA methylation patterns. Moreover, granulosa cells and cumulus cells protect the oocyte when they are exposed to a high fatty acid environment via reducing the quantity of NEFAs that oocyte-exposed either through lipid droplets storage or oxidization. Besides, the granulosa cells can produce and deliver some anti-inflammatory factors to cumulus cells and oocytes through the bidirectional communication. Therefore, the anti-inflammatory response exists in blastocyst originated from the high level of NEFAs-exposed oocyte.

An elevated level of NEFA is one of the metabolic issues that impair oocyte quality and development, and the resulting defects may be retained in the following generations. In this study, we summarized the common and specific effects of NEFA on ovarian cells in humans, cows, mice, and pigs (Fig. 1). Fatty acids can be used as substrate to generate energy to maintain biological functions, and their storage and oxidation are important for oocyte and embryo development. Humans and domestic animals are both affected by abnormal FA levels which are regulated by the nutrition status. The bovine model is the most studied, and studies of humans are limited because of ethical problems. Nutrition-related fertility problems in pigs are less studied. More studies should focus on pigs, not only because they have physiological, nutritional, genomic homologies, and preimplantation development similarities compared to humans (Madeja *et al.* 2019) but also because the same problems also exist in this species, and the related mechanisms should be identified.

## Declaration of interest

The authors declare that there is no conflict of interest that could be perceived as prejudicing the impartiality of this review.

## Funding

This study was supported by the Natural Sciences and Engineering Research Council of Canada (NSERC grant number 445230-12). Fonds de recherche du Québec – Nature et technologies (FRQNT) provided a fellowship to Meihong Shi.

## Author contribution statement

Meihong Shi designed and wrote the paper, and Marc-André Sirard designed and revised the paper.
